# Investigation of Peak Pressure Index Parameters for People with Spinal Cord Injury Using Wheelchair Tilt-in-Space and Recline: Methodology and Preliminary Report

**DOI:** 10.1155/2014/508583

**Published:** 2014-06-26

**Authors:** Chi-Wen Lung, Tim D. Yang, Barbara A. Crane, Jeannette Elliott, Brad E. Dicianno, Yih-Kuen Jan

**Affiliations:** ^1^Rehabilitation Engineering Laboratory, Department of Kinesiology and Community Health, University of Illinois at Urbana-Champaign, 1206 South Fourth Street, MC-588, Champaign, IL 61820, USA; ^2^Department of Creative Product Design, Asia University, Taichung 41354, Taiwan; ^3^Department of Rehabilitation Sciences, University of Hartford, West Hartford, CT 06117, USA; ^4^Division of Disability Resources and Educational Services, University of Illinois at Urbana-Champaign, Champaign, IL 61820, USA; ^5^Department of Physical Medicine and Rehabilitation, University of Pittsburgh Medical Center, Pittsburgh, PA 15206, USA

## Abstract

The purpose of this study was to determine the effect of the sensel window's location and size when calculating the peak pressure index (PPI) of pressure mapping with varying degrees of wheelchair tilt-in-space (tilt) and recline in people with spinal cord injury (SCI). Thirteen power wheelchair users were recruited into this study. Six combinations of wheelchair tilt (15°, 25°, and 35°) and recline (10° and 30°) were used by the participants in random order. Displacements of peak pressure and center of pressure were extracted from the left side of the mapping system. Normalized PPI was computed for three sensel window dimensions (3 sensels × 3 sensels, 5 × 5, and 7 × 7). At least 3.33 cm of Euclidean displacement of peak pressures was observed in the tilt and recline. For every tilt angle, peak pressure displacement was not significantly different between 10° and 30° recline, while center of pressure displacement was significantly different (*P* < .05). For each recline angle, peak pressure displacement was not significantly different between pairs of 15°, 25°, and 35° tilt, while center of pressure displacement was significantly different between 15° versus 35° and 25° versus 35°. Our study showed that peak pressure displacement occurs in response to wheelchair tilt and recline, suggesting that the selected sensel window locations used to calculate PPI should be adjusted during changes in wheelchair configuration.

## 1. Introduction

Sitting-acquired pressure ulcers result from loading-induced soft tissue necrosis [1–3]. Pressure ulcers are both common (up to 85% lifetime incidence) and chronic (up to 70% recurrence) among people with spinal cord injury (SCI), largely due to the heightened pressure ulcer risk associated with diminished capacities to sense pain and to perform weight shifts [4, 5]. In the United States, treatment for pressure ulcers has been estimated to cost $1.2 billion annually, accounting for one-quarter of the total cost of SCI care [6].

Although pressure ulcer etiology is multifactorial, leading hypotheses are that tissue ischemia and tissue deformation are associated with the precursory tissue necrosis [1–3]. The former proposes the fact that mechanical loading prevents arterial vessels from resupplying tissues with oxygen and nutrients, leading to tissue ischemia and ultimately tissue necrosis. The latter proposes the fact that mechanical loading causes compressive and shearing deformation at the cellular level, leading to individual cell deaths and ultimately tissue necrosis. The apparent link between tissue necrosis and mechanical loading has prompted the development and evaluation of seating support surfaces in terms of optimizing seating interface pressure distributions [7–11]. Empirically, there is evidence linking increased mechanical loading with increased pressure ulcer incidence in elderly wheelchair users [12]. Clinically, best-practice guidelines recommend routinely performing pressure-relieving maneuvers to further manage pressure ulcer risk [13–15].

To evaluate seating pressure distributions, interface pressure mapping (IPM) is commonly used to measure the normal forces at the seating interface. Brienza et al. [12] used IPM in a randomized clinical trial to explore the relationship between seating interface pressure and pressure ulcer incidence. Among 32 elderly wheelchair users, seating interface pressure was significantly higher among those who developed pressure ulcers during the trial compared to those who did not. Others have used IPM to assess tissue viability in response to postural interventions among people with SCI [16]. Various IPM metrics have been reported in the literature, including average pressure, peak pressure, peak pressure index, peak pressure gradient, peak pressure ratio, and dispersion index [2, 17, 18]. For any given IPM metric, there is always a tradeoff between reliability and repeatability versus descriptiveness. For example, while peak pressure (i.e., the peak sensel value) provides very precise information about ischial loading, it is relatively unreliable and unrepeatable; and while average pressure (i.e., the sensel average throughout the entire contact area) is relatively reliable and repeatable, it masks precise information about ischial loading [19]. In the literature, peak pressure index (PPI, the sensel average within a 10 cm^2^ window of the peak pressure sensel) has been reported to strike a reasonable balance during cushion bench tests and tests of interface pressure with manual wheelchair seat angle changes [18].

**Figure 1:**
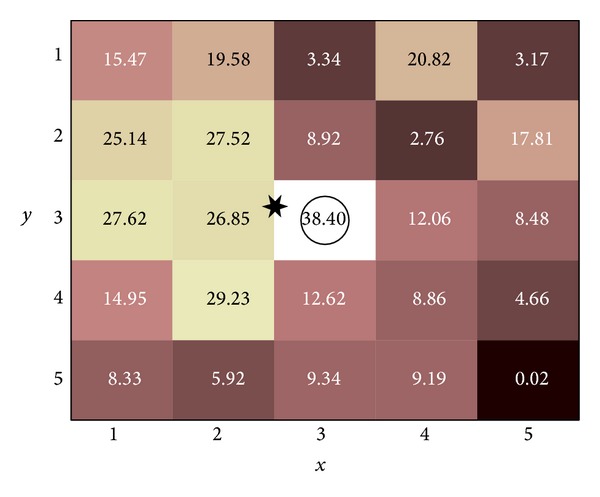
A sample 5 × 5 sensel window. The peak pressure refers to the maximal sensel value in the window. The peak pressure index refers to the average sensel value in the window. The center of pressure coordinate refers to the summation of the product of each sensel's pressure value with its grid coordinate, divided by the total pressure sum; that is, (∑_*i*=1_^*n*^ *P*_*i*_ *x*_*i*_/∑_*i*=1_^*n*^ *P*_*i*_, ∑_*i*=1_^*n*^ *P*_*i*_ *y*_*i*_/∑_*i*=1_^*n*^ *P*_*i*_), where *n* is the number of sensels, *P*_*i*_ is the pressure of the *i*th sensel, and *x*_*i*_ and *y*_*i*_ are the *x* and *y* coordinates of the *i*th sensel. For this sample, the peak pressure (circled) is 38.40 mm Hg, the peak pressure index is 14.44 mm Hg, and the center of pressure coordinate (starred) is at *x* = 2.53, *y* = 2.80.

The purpose of this study was to investigate the selection of two sensel window parameters of the PPI metric in response to wheelchair tilt-in-space (tilt) and recline. Recent studies have begun using PPI to assess the effectiveness of tilt and recline on relieving seating interface pressure [20–22]. Due to seating perturbations during dynamic seating conditions, special care may be needed when analyzing and interpreting seating interface pressures. Studies have reported 1.5 cm and 6 cm of sliding displacement during various dynamic seating tests, including tilt, recline, and forward flexion maneuvers [19, 23–26]. Depending on the analyzed region of interest, these interface displacements may significantly alter computed seating pressure metrics, such as PPI. In addition, secondary metrics, such as center of pressure, may be affected. Center of pressure refers to the coordinates obtained by summing the product of each sensel's pressure value with its grid coordinate and dividing the result by the total pressure sum; that is, (∑_*i*=1_^*n*^ *P*_*i*_ *x*_*i*_/∑_*i*=1_^*n*^ *P*_*i*_, ∑_*i*=1_^*n*^ *P*_*i*_ *y*_*i*_/∑_*i*=1_^*n*^ *P*_*i*_), where *n* was the number of sensels, *P*_*i*_ was the pressure of the *i*th sensel, and *x*_*i*_ and *y*_*i*_ were the *x* and *y* coordinates of the *i*th sensel [27]. Sonenblum and Sprigle [22] explored center of pressure displacement and found a significant displacement when tilting 15° beyond the upright posture. We investigated the changes in PPI and center of pressure in response to two calculation parameters: sensel window location and sensel window size. The sensel window refers to the group of sensels considered by the PPI computation. The sensel window dimension refers to the number of sensels along each side (e.g., 3 sensels × 3 sensels); the sensel window size refers to the 2-dimensional area occupied by the window (e.g., 10 cm^2^); and the sensel window location refers to its placement within the pressure value grid (Figure 1). We explored the sensel window location via calculating the Euclidean displacements of peak pressure and center of pressure in response to tilt and recline, and we explored the sensel window size via comparing normalized PPI in response to different sensel window sizes.

## 2. Method

This study used an intervention and outcomes research design with repeated measures.

### 2.1. Participants

We enrolled 13 wheelchair users with SCI into the study. Participants were recruited via research flyers and referrals from a local rehabilitation hospital. Inclusion criteria included having traumatic SCI between the levels of C4 and T5, being at least 6 months after spinal injury, using a power wheelchair as the primary means of mobility and using a wheelchair seat width of 43 cm (17 in) to 53 cm (21 in). Exclusion criteria included having diseases that may affect cardiovascular function, diagnosed skeletal deformities (e.g., scoliosis, pelvic obliquity, and hip and knee contracture), or active pressure ulcers. All participants provided informed consent to this study, which was approved by the Institutional Review Board at the University of Oklahoma Health Sciences Center. The demographic data of the participants were as follows (values are mean ± SD): age 36.2 ± 10.0 years, body weight 77.4 ± 17.9 kg, body mass index 24.6 ± 4.6 kg/m^2^, and duration of injury 5.8 ± 5.9 years. The 13 wheelchair users included 4 women and 9 men: 3 African Americans, 1 Asian American, 1 multirace American, and 8 Caucasian Americans. Four participants had sensory complete injury (American Spinal Injury Association Impairment Scale (AIS) A), 2 participants had motor complete injury (AIS B), and 7 participants had incomplete injury (AIS C). All participants used a power wheelchair for mobility.

### 2.2. Apparatus

Seating interface pressures were recorded with an interface pressure mat (CONFORMat 5330, Tekscan, Boston, MA). The mat contains a 32 × 32 grid-based array of extremely thin, flexible tactile sensors. The sensor array can measure an area up to 47.1 cm × 47.1 cm with each sensel measuring approximately 1.47 cm × 1.47 cm. The mat system was calibrated before each subject's data collection, based on the manufacturer's guidelines.

**Figure 2:**
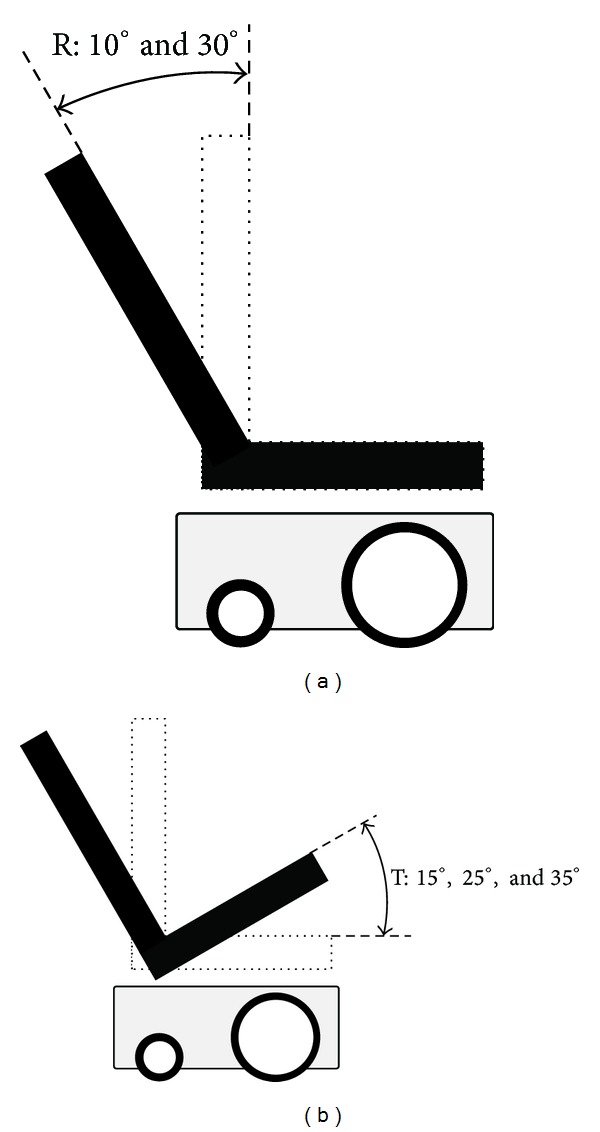
Configurations of wheelchair (a) recline and (b) tilt-in-space. R: recline; T: tilt-in-space.

A power wheelchair (C300 Corpus, Permobil, Lebanon, TN) with tilt and recline seat positioning functions was used in this study. The seat width was 48 cm (19 in). A standard high-density precontoured foam seat cushion (Corpus seating system, Permobil, Lebanon, TN) was used in this study. Tilt was defined as “a change of seat angle orientation in relation to the ground while maintaining the seat to back angle” [4, 5]. In this study, tilt referred only to the backward direction. Wheelchair recline was defined as “a change in seat to back angle while maintaining a constant seat angle with respect to the ground” [4, 5]. Configurations of tilt and recline are shown in Figure 2 and also described in our previous studies with the exception of recline measurements [14, 28, 29]. Previously, we reported recline measurements as the angle between the seat and the back support. In keeping with current clinical practice, we have modified our recline measurements to represent the sagittal angle of the back support from the vertical [30]. That is to say, our previously reported recline angles of 100° and 120° are now reported as 10° and 30°, respectively, although the actual recline configurations have not changed. Two digital angle gauges (WR300, Wixey, online-based company) were used to measure the tilt and recline angles.

### 2.3. Protocol

The wheelchair configuration protocol has been explained in our previous studies [14, 28, 29]. These studies were conducted in parallel to assess skin perfusion response to tilt and recline. The same subjects and protocols were used in these parallel studies. The tilt and recline protocol has been summarized in Table 1.

### 2.4. Procedure

Before the experiment, participants first provided informed consent. They were then asked to empty their bladders and to remain in the laboratory for 30 min to acclimatize to room temperature (23°C ±2°C). During the acclimatization period, the IPM mat was placed atop the standard precontoured seat cushion of the test power wheelchair. Upon completion of the acclimatization period, the participant was transferred to a mat table to affix a laser Doppler flowmetry sensor, which was used to measure skin perfusion for our parallel study regarding skin blood flow response to tilt and recline [14]. The participant was then transferred to the test power wheelchair, which contained an IPM mat at the interface between the cushion and buttocks. The participant was asked to place his or her hands in the lap and to sit as far back as possible while remaining comfortable. The foot support was adjusted to ensure that the femurs were parallel to the floor. The ischial tuberosities and coccygeal areas were palpated to ensure that they were positioned over the IPM mat. After a 6 min settling period to reduce the effects of creep [31], the IPM mat was calibrated for the given participant to minimize drift and hysteresis according to the manufacturer's procedure.

Each experiment began with a washout configuration of 35° tilt and 30° recline. During the experiment, IPM samples were recorded at 10 Hz, and the range of acceptable angles was ±3° of the desired angle. To minimize operator effects, the same research assistant performed the tilt and recline adjustments for all experiments in this study. To minimize sequence effects, we used a balanced design with randomized testing protocols. To minimize carryover effects, we ended every testing condition with a washout configuration of 35° tilt and 30° recline, which also served to provide a recovery period of maximal pressure relief to the participant at least every 15 min, which satisfies the recommended pressure ulcer prevention guidelines [14]. Each participant spent roughly 100 min completing the entire protocol.

### 2.5. Data Analysis

A bicubic spline was first applied to the data prior to the metric calculations. Computations were performed using MATLAB 2012a (Mathworks, Natick, MA). For the displacement analysis, the independent variable was the seating orientation, which included six combinations of tilt (15°, 25°, and 35°) and recline (10° and 30°). The dependent variable was the displacement of either peak pressure or center of pressure among the various seating configurations. We performed two sets of comparisons. First, we grouped the recline angles to assess the effect of tilt on displacement. One-way analysis of variance (ANOVA) with Fisher's least significant difference (LSD) post hoc tests were used to compare pairwise displacement differentials between 15°, 25°, and 35° tilt for the two tested recline angles. Second, we grouped the tilt angles to assess the effect of recline on displacement. Paired samples* t*-tests were used to compare the displacement differential between 10° and 30° recline for the three tilt angles. We considered using Bonferroni corrections; however, because of the high number of measures, low number of participants, and exploratory nature of the pilot study, we decided not to use these corrections due to the increased likelihood of committing a type II error, which was not acceptable for an exploratory study [32].

For the sensel window size analysis, the independent variable was the sensel window area, which included three configurations: 3 sensels × 3 sensels (19.45 cm^2^), 5 × 5 (54.02 cm^2^), and 7 × 7 (105.88 cm^2^) (Figure 1). We chose these sensel window dimensions primarily for two reasons. First, a 2 × 2 sensel window would have fallen under the recommended 9-10 cm^2^ area (i.e., estimated contact area of the ischial tuberosity) of the PPI metric [9, 18]. Second, the use of odd and square dimensions allowed us to consistently situate the peak sensel in the same location (i.e., the center) of the sensel window. The dependent variable was the peak pressure average within the sensel window. Seating pressures were averaged across each 5 min testing period. Values during each tilted and reclined period were normalized to their corresponding baseline values (Table 1). Because up to 30 sec were needed to complete the tilt and recline angle adjustments, seating pressures obtained during the first 30 sec of each 5 min tilted and reclined period were excluded from analysis. A one-way ANOVA with Fisher's LSD post hoc test was used to examine the efficacy of repeated measures between the 3 × 3, 5 × 5, and 7 × 7 sensel window dimensions. All statistical tests were performed using SPSS 22 (IBM, Somers, NY) at the significance level of .05.

## 3. Results

### 3.1. Displacement

Overall, peak pressure displacement between the upright and testing configurations ranged from 3.3 cm to 6.6 cm. Center of pressure displacement between the upright and testing configurations ranged from 0.6 cm to 1.7 cm.

#### 3.1.1. Tilt Angle Effect

**Figure 3:**
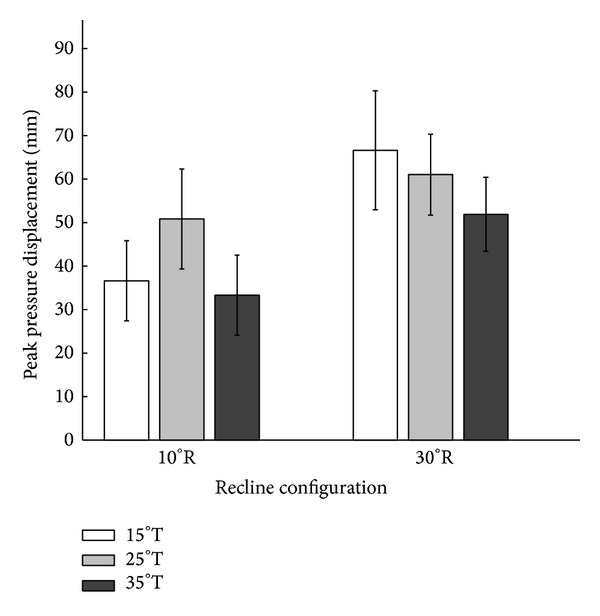
Pairwise comparisons of peak pressure displacement in the left side of the seat in response to six combinations of wheelchair tilt-in-space (15°, 25°, and 35°) and recline (10° and 30°). Data are shown as mean ± SE. R: recline; T: tilt-in-space.

Pairwise comparisons of peak pressure displacement did not reveal significant differences between 15°, 25°, and 35° tilt for each of the two tested recline angles (Figure 3).

**Figure 4:**
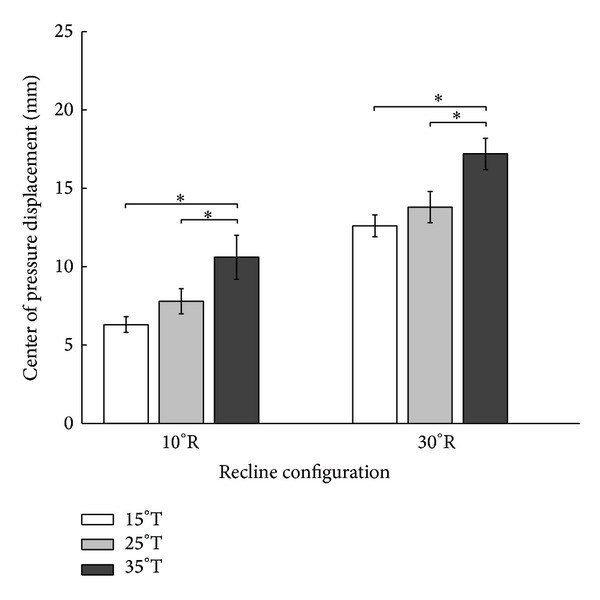
Pairwise comparisons of center of pressure displacement in the left side of the seat in response to six combinations of wheelchair tilt-in-space (15°, 25°, and 35°) and recline (10° and 30°). Data are shown as mean ± SE. R: recline; T: tilt-in-space; ∗a significant difference (*P* < .05).

At 10° recline, pairwise comparisons of center of pressure displacement revealed significant differences between the following pairs of tilt angles: 15° versus 35° and 25° versus 35° (*P* < .05; see Figure 4). At 30° recline, pairwise comparisons of center of pressure displacement revealed significant differences between the same pairs of tilt angles: 15° versus 35° and 25° versus 35° (*P* < .05; Figure 4).

#### 3.1.2. Recline Angle Effect

**Figure 5:**
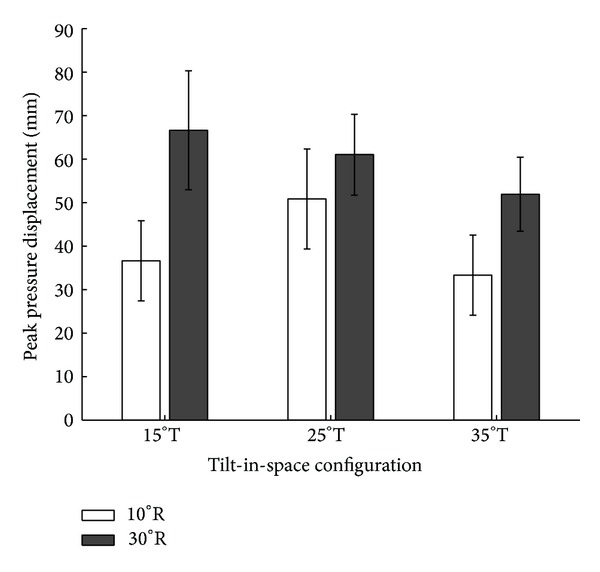
Comparisons of peak pressure displacement in the left side of the seat for three configurations of wheelchair tilt-in-space (15°, 25°, and 35°) in response to recline (10° and 30°). Data are shown as mean ± SE. R: recline; T: tilt-in-space.

Comparisons of peak pressure displacement did not reveal significant differences between 10° and 30° recline for each of the three tilt angles (Figure 5).

**Figure 6:**
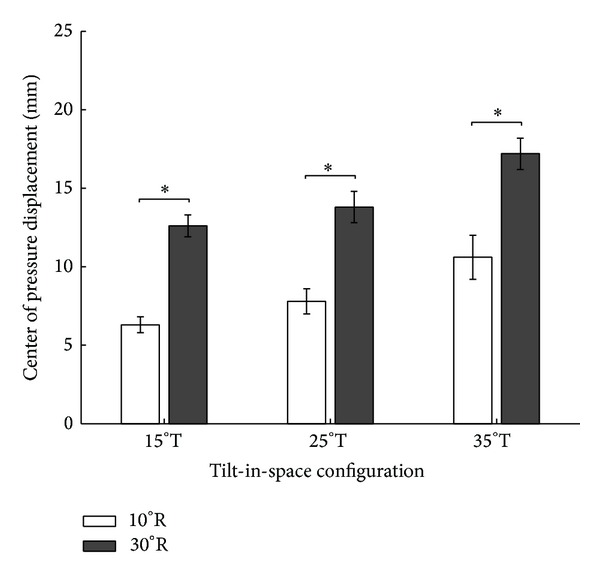
Comparisons of center of pressure displacement in the left side of the seat for three configurations of wheelchair tilt-in-space (15°, 25°, and 35°) in response to recline (10° and 30°). Data are shown as mean ± SE. R: recline; T: tilt-in-space; ∗a significant difference (*P* < .05).

At 15° tilt, center of pressure displacement was significantly different between 10° and 30° recline (*P* < .05; see Figure 6). At 25° tilt, center of pressure displacement was significantly different between 10° and 30° recline (*P* < .05; see Figure 6). At 35° tilt, center of pressure displacement was significantly different between 10° and 30° recline (*P* < .05; Figure 6).

### 3.2. Sensel Window Size

**Figure 7:**
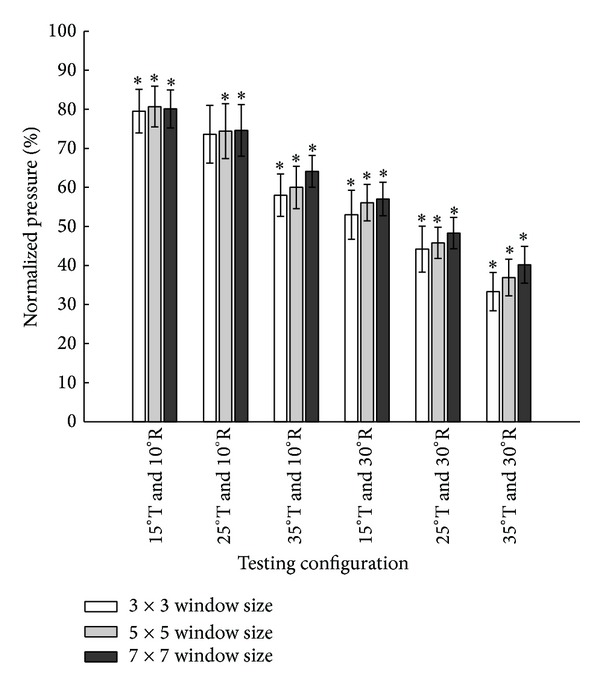
Comparisons of three sensel window sizes (3 × 3, 5 × 5, and 7 × 7) of normalized ischial pressure in response to combinations of wheelchair tilt-in-space (15°, 25°, and 35°) and recline (10° and 30°). Data are shown as mean ± SE. R: recline; T: tilt-in-space; ∗a significant difference compared to the upright sitting position (*P* < .05).

Comparisons of normalized PPI calculations among the three sensel window sizes for each wheelchair configuration revealed no significant differences.  Figure 7 shows the normalized PPI in each wheelchair configuration for all three sensel window sizes. The statistical significance of normalized PPI versus the upright seating position was identical across all sensel window areas, except under one testing condition (25° tilt and 10° recline), in which the 5 × 5 and 7 × 7 sensel window sizes yielded significantly different PPI values while comparisons with the 3 × 3 window did not.

## 4. Discussion

Our study confirmed the occurrence of peak pressure and center of pressure displacement during combinations of tilt and recline. Although sliding displacement has been reported in the literature as a clinical challenge of wheelchair seating [33], few studies have investigated its effects on clinical and research applications. Cooper et al. [24] used motion capture cameras to measure sliding displacement during wheelchair sit-to-stand and recline operations. For hybrid and air cushions, they reported approximately 1.5 cm and 3.5 cm, respectively, of thigh displacement during recline. However, their testing did not include participants with SCI (rather, anthropometric test dummies) or standard foam cushions (rather, hybrid and air cushions). Hobson and Tooms [19, 23] used radiography to investigate pelvic movement across various body postures in people with SCI. When comparing a slight back support recline with 30° forward trunk flexion, they found the ischial tuberosity to be displaced by an average of 2.7 cm; and across all tested postures, they reported an average shift of approximately 4 cm. However, their experiment did not include IPM data to correlate with the radiographic data. Aissaoui et al. [25] quantified the sliding displacement of the buttock along the seat plane during repositioning and found approximately 6 cm of horizontal sliding at 30° tilt and 30° recline. However, their study only included participants without disability.

In our study, we recruited people with SCI and tested combinations of tilt and recline while seated on a precontoured foam cushion. Instead of relying on external measures of displacement, we examined the direct displacement of peak pressures at the buttock-cushion interface. Even at the lowest observed levels of sliding, we recorded peak pressure displacements of more than 3 cm. At the highest extreme, we observed nearly 7 cm of peak pressure displacement. These displacements indicate that when analyzing IPM data of people with SCI, the PPI sensel window should be moved in response to tilt and recline maneuvers. Otherwise, the peak pressures would frequently shift outside of the traditional PPI sensel window size of 10 cm^2^.

Because pairwise comparisons of peak pressure displacement did not reveal significant differences, peak pressure displacement may be relatively insensitive to varying wheelchair seating configurations. That is to say, for a given tilt angle, changing the recline angle does not appear to produce significantly different displacements from one another and vice versa. However, this perhaps does not adequately represent the effect of wheelchair seating configuration on IPM measures. The effect of tilt and recline adjustments becomes more pronounced when we consider secondary metrics that have been computed from the raw IPM values, such as center of pressure. Among all three tilt angles, displacements of the computed centers of pressure were significantly different between the two tested recline angles. For each recline angle, center of pressure displacements was significantly different between two of the three tilt angle pairs. Thus, while raw peak pressure displacement was not significantly sensitive to varying wheelchair configurations, secondary metrics computed from the raw IPM values appear to be significantly affected.

Few studies have investigated center of pressure displacement in response to varying wheelchair configurations. One study used an instrumented simulator chair to mechanically actuate postural changes in 8 people without disability [34]. The study measured, among other metrics, seating interface pressure response to postural change and found center of pressure displacement to be a sensitive measure in response to wheelchair recline and pelvic rotation. Sonenblum and Sprigle [22] assessed 11 participants with SCI and found that the center of pressure was displaced significantly at 15° tilt compared to the upright position. Our study augments the previous reports of center of pressure displacement, providing center of pressure displacement in response to six wheelchair configurations among people with SCI.

In particular, the sensitivity of the center of pressure displacement metric is promising because it may be useful in the future to provide insight into currently unexplained physiological phenomena. Our previous work indicated that combinations of tilt and recline angles with lower than 25° tilt and 30° recline may not be sufficient to stimulate an effective skin perfusion response [14]. However, those findings are not universally representative of every person with SCI. We are optimistic that specific characteristics of center of pressure displacement may be helpful in understanding individual differences in skin perfusion response to tilt and recline. For example, two people may exhibit similar interface pressure measures at the ischial tuberosity, but significantly different perfusion responses. Increased center of pressure displacement may be an indicator for increased biomechanical changes in local soft tissues, stimulating higher microcirculation in those tissues. Further work is needed to explore whether lower-than-expected displacements in center of pressure displacement can be correlated with lower-than-expected skin perfusion measures in response to tilt and recline.

Sliding displacement characteristics may also be relevant to skin perfusion response in terms of the associated shear forces, which are known to contribute to internal tissue strain and blood vessel occlusion [35]. Hobson [19] found a 25% increase of shear forces in response to 30° recline and the elimination of shear forces in response to 20° tilt. Although shear forces are of significant interest to pressure ulcer researchers, shear forces are difficult to measure without significantly altering the interface properties. While sliding displacement is not a direct measure of shear, it may still serve as a useful surrogate measure of shear forces at the seating interface. Because interface pressure displacement can be measured with IPM systems that are commercially available and do not significantly alter the seating interface, it may be an appropriate proxy measure for shear force response to tilt and recline.

**Figure 8:**
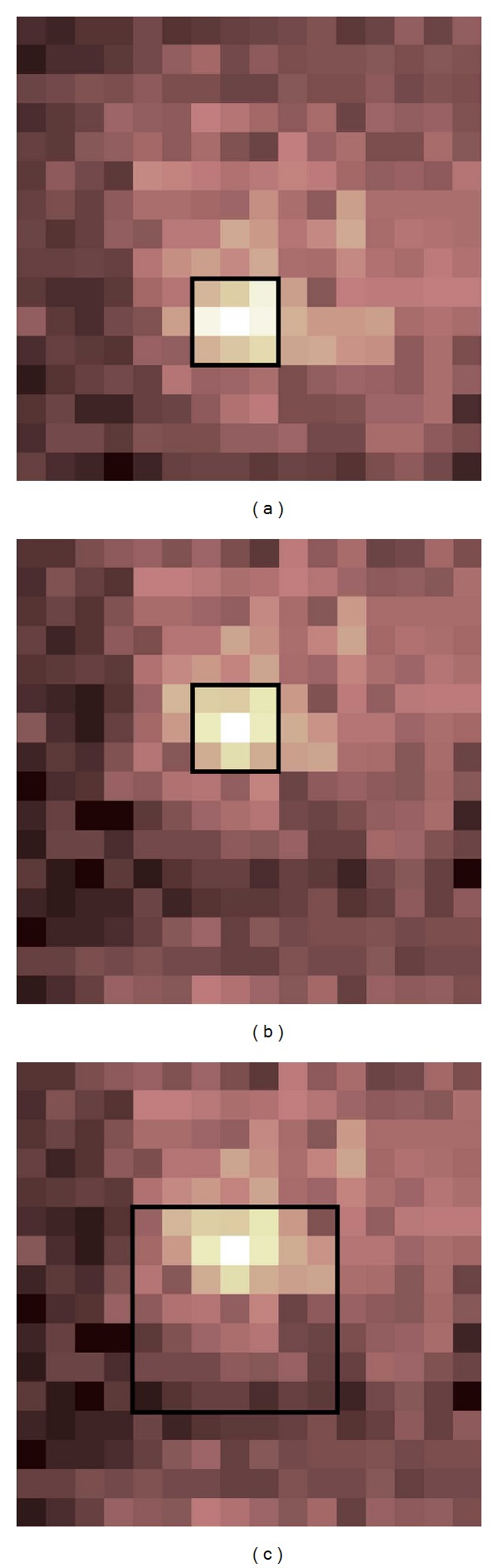
For IPM analysis, sliding displacement complicates the analysis. Two solutions are immediately apparent: move the sensel window location (a and b) or expand the sensel window area (a and c).

For IPM analysis, sliding displacement complicates the analysis. Two solutions are immediately apparent: move the sensel window location (Figure 8(b)) or expand the sensel window area (Figure 8(c)). Although one study customized a sensor array (7 cm diameter) of interface pressure sensors and directly attached it to the buttock skin [22], a more practical solution for general clinical and research usage would be to adjust the sensel window parameters during IPM analysis. In our study, we adjusted the sensel window location for each tilt and recline maneuver. Sensel window dimensions were then increased to examine the effect of the sensel window area on the PPI metric. Preliminary findings indicate that if the sensel window location is adjusted appropriately during dynamic seating, the PPI metric does not appear to be sensitive to varying sensel window sizes. Despite observed peak pressure displacements and significant center of pressure displacements, only one of the sensel window sizes within one testing condition differed statistically in normalized PPI. In all other conditions, the statistical significance of normalized PPI was not affected by sensel window size. Moreover, the one-way ANOVA did not reveal any significant pairwise differences in normalized PPI amongst the three sensel window sizes. These findings suggest that as long as sensel window locations are appropriately selected following tilt and recline changes, larger sensel window areas may not be necessary.

Our study was designed with clearly demarcated time periods for each wheelchair configuration. Thus, we afforded the benefit of distinct cutoff points to indicate when the sensel window should be moved. For more generalized settings in which the seating posture may be less structured and more dynamic, it may not be feasible to know when the sensel window location should be moved. In such cases, we propose two options. First, it may be preferable to perform a PPI time series analysis. That is, PPI would be computed at each time point, and the sensel window location would be dynamically selected for each frame. Further work is needed to determine the reliability and repeatability of this method. Second, the sensel window size could be enlarged. That is, the sensel window location would be maintained statically throughout the entire data set, but the increased sensel window size would be able to accommodate unpredictable displacements. Further work is needed to determine an appropriate sensel window size and whether the noncentered peak concentrations would significantly affect PPI calculations.

There are limitations of this study. First, a laser Doppler flowmetry sensor was attached at the site of the ischial tuberosity as part of our parallel study regarding skin perfusion response to tilt and recline [14]. However, the probe was small and thin, and we analyzed normalized pressure measures, instead of pure magnitudes. While there may be some residual effects from the attached probe, we felt it was a justifiable limitation to facilitate our long-term goal of integrating skin perfusion measures with interface pressure measures to develop a comprehensive assessment of wheelchair configurations. Second, our inclusion criteria specified people who used a seat width between 43 cm and 53 cm. In future work, multiple seat widths will be tested to accommodate a broader demographic of participants. Third, we only recruited 13 wheelchair users with SCI. This was initially a feasibility study to examine whether our protocols could be used to assess the efficacy of tilt and recline. In future work, a larger sample size using this protocol should be conducted to verify our findings.

## 5. Conclusions

Our study demonstrated the existence of peak pressure displacement and center of pressure displacement during tilt and recline maneuvers. Thus, the PPI sensel window location should be adjusted during position changes. Preliminary evidence also indicates that if the sensel window location is adjusted appropriately, increasing the sensel window dimensions from 3 × 3 up to 7 × 7 does not have a significant effect on the PPI metric. In future work, we hope to establish peak pressure displacement as a surrogate measure for shear force and center of pressure displacement as an indicator for individual differences in skin perfusion response to tilt and recline.

## Figures and Tables

**Table 1:** Repeated measures before and after trial design.

Randomized configurations	Baseline condition (5 min)	Testing condition (5 min)	Washout condition (5 min)
1	0°T & 0°R	15°T & 10°R	35°T & 30°R
2	0°T & 0°R	25°T & 10°R	35°T & 30°R
3	0°T & 0°R	35°T & 10°R	35°T & 30°R
4	0°T & 0°R	15°T & 30°R	35°T & 30°R
5	0°T & 0°R	25°T & 30°R	35°T & 30°R
6	0°T & 0°R	35°T & 30°R	35°T & 30°R

R: recline; T: tilt-in-space.

## Abbreviations

ANOVA: Analysis of variance
IPM: Interface pressure mapping
PPI: Peak pressure index
SCI: Spinal cord injury.

## References

[1] Olesen CG, de Zee M, Rasmussen J (2010). Missing links in pressure ulcer research—an interdisciplinary overview. *Journal of Applied Physiology*.

[2] Loerakker S, Manders E, Strijkers GJ (2011). The effects of deformation, ischemia, and reperfusion on the development of muscle damage during prolonged loading. *Journal of Applied Physiology*.

[3] Liao F, Burns S, Jan YK (2013). Skin blood flow dynamics and its role in pressure ulcers. *Journal of Tissue Viability*.

[4] Jan YK, Brienza DM (2006). Technology for pressure ulcer prevention. *Topics in Spinal Cord Injury Rehabilitation*.

[5] Dicianno BE, Arva J, Lieberman JM (2009). RESNA position on the application of tilt, recline, and elevating legrests for wheelchairs. *Assistive Technology*.

[6] Byrne DW, Salzberg CA (1996). Major risk factors for pressure ulcers in the spinal cord disabled: a literature review. *Spinal Cord*.

[7] Sprigle S, Schuch JZ (1993). Using seat contour measurements during seating evaluations of individuals with SCI. *Assistive Technology*.

[8] Burns SP, Betz KL (1999). Seating pressures with conventional and dynamic wheelchair cushions in tetraplegia. *Archives of Physical Medicine and Rehabilitation*.

[9] Sprigle S, Dunlop W, Press L (2003). Reliability of Bench Tests of Interface Pressure. *Assistive Technology*.

[10] Akins JS, Karg PE, Brienza DM (2011). Interface shear and pressure characteristics of wheelchair seat cushions. *Journal of Rehabilitation Research and Development*.

[11] Metring NL, Gaspar MIFAS, Mateus-Vasconcelos ECL, Gomes MM, De Abreu DCC (2012). Influence of different types of seat cushions on the static sitting posture in individuals with spinal cord injury. *Spinal Cord*.

[12] Brienza DM, Karg PE, Geyer MJ, Kelsey S, Trefler E (2001). The relationship between pressure ulcer incidence and buttock-seat cushion interface pressure in at-risk elderly wheelchair users. *Archives of Physical Medicine and Rehabilitation*.

[13] Makhsous M, Priebe M, Bankard J (2007). Measuring tissue perfusion during pressure relief maneuvers: insights into preventing pressure ulcers. *Journal of Spinal Cord Medicine*.

[14] Jan YK, Jones MA, Rabadi MH, Foreman RD, Thiessen A (2010). Effect of wheelchair tilt-in-space and recline angles on skin perfusion over the ischial tuberosity in people with spinal cord injury. *Archives of Physical Medicine and Rehabilitation*.

[15] Jan YK, Brienza DM, Boninger ML, Brenes G (2011). Comparison of skin perfusion response with alternating and constant pressures in people with spinal cord injury. *Spinal Cord*.

[16] Wu GA, Lombardo L, Triolo RJ, Bogie KM (2013). The effects of combined trunk and gluteal neuromuscular electrical stimulation on posture and tissue health in spinal cord injury. *PM and R*.

[17] Apatsidis DP, Solomonidis SE, Michael SM (2002). Pressure distribution at the seating interface of custom-molded wheelchair seats: effect of various materials. *Archives of Physical Medicine and Rehabilitation*.

[18] Maurer CL, Sprigle S (2004). Effect of seat inclination on seated pressures of individuals with spinal cord injury. *Physical Therapy*.

[19] Hobson DA (1992). Comparative effects of posture on pressure and shear at the body-seat interface. *Journal of Rehabilitation Research and Development*.

[20] Sprigle S, Maurer C, Sorenblum SE (2010). Load redistribution in variable position wheelchairs in people with spinal cord injury. *Journal of Spinal Cord Medicine*.

[21] Giesbrecht EM, Ethans KD, Staley D (2011). Measuring the effect of incremental angles of wheelchair tilt on interface pressure among individuals with spinal cord injury. *Spinal Cord*.

[22] Sonenblum SE, Sprigle SH (2011). The impact of tilting on blood flow and localized tissue loading. *Journal of Tissue Viability*.

[23] Hobson DA, Tooms RE (1992). Seated lumbar/pelvic alignment—a comparison between spinal cord-injured and noninjured groups. *Spine*.

[24] Cooper RA, Dvorznak MJ, Rentschler AJ, Boninger ML (2000). Displacement between the seating surface and hybrid test dummy during transitions with a variable configuration wheelchair: a technical note. *Journal of Rehabilitation Research and Development*.

[25] Aissaoui R, Lacoste M, Dansereau J (2001). Analysis of sliding and pressure distribution during a repositioning of persons in a simulator chair. *IEEE Transactions on Neural Systems and Rehabilitation Engineering*.

[26] Tam EW, Mak AF, Lam WN, Evans JH, Chow YY (2003). Pelvic movement and interface pressure distribution during manual wheelchair propulsion. *Archives of Physical Medicine and Rehabilitation*.

[27] Han TR, Paik NJ, Im MS (1999). Quantification of the path of center of pressure (COP) using an F-scan in-shoe transducer. *Gait and Posture*.

[28] Jan YK, Crane BA, Liao F, Woods JA, Ennis WJ (2013). Comparison of muscle and skin perfusion over the ischial tuberosities in response to wheelchair tilt-in-space and recline angles in people with spinal cord injury. *Archives of Physical Medicine and Rehabilitation*.

[29] Jan YK, Crane BA (2013). Wheelchair tilt-in-space and recline does not reduce sacral skin perfusion as changing from the upright to the tilted and reclined position in people with spinal cord injury. *Archives of Physical Medicine and Rehabilitation*.

[30] Waugh K, Crane B (2013). *A Clinical Application Guide to Standardized Wheelchair Seating Measures of the Body and Seating Support Surfaces*.

[31] Stinson M, Porter A, Eakin P (2002). Measuring interface pressure: a laboratory-based investigation into the effects of repositioning and sitting. *The American Journal of Occupational Therapy*.

[32] Perneger TV (1998). What’s wrong with Bonferroni adjustments. *British Medical Journal*.

[33] Pope PM (1985). A study of instability in relation to posture in the wheelchair. *Physiotherapy*.

[34] van Geffen P, Reenalda J, Veltink PH, Koopman BF (2008). Effects of sagittal postural adjustments on seat reaction load. *Journal of Biomechanics*.

[35] Jan YK, Brienza DM, Gefen A (2009). Tissue mechanics and blood flow factors in pressure ulcers of people with spinal cord injury. *The pathomechanics of tissue injury and disease, and the mechanophysiology of healing*.

